# Female-Bias in a Long-Term Study of a Species with Temperature-Dependent Sex Determination: Monitoring Sex Ratios for Climate Change Research

**DOI:** 10.1371/journal.pone.0160911

**Published:** 2016-08-31

**Authors:** Joanne Braun McNeill, Larisa Avens, April Goodman Hall, Lisa R. Goshe, Craig A. Harms, David W. Owens

**Affiliations:** 1 Southeast Fisheries Science Center, National Marine Fisheries Service, National Oceanic and Atmospheric Administration, Beaufort, North Carolina, United States of America; 2 Center for Marine Sciences and Technology, College of Veterinary Medicine, North Carolina State University, Morehead City, North Carolina, United States of America; 3 University of Charleston, South Carolina at the College of Charleston, Charleston, South Carolina, United States of America; Deakin University, AUSTRALIA

## Abstract

Alterations have occurred and continue to manifest in the Earth’s biota as a result of climate change. Animals exhibiting temperature dependent sex determination (TSD), including sea turtles, are perhaps most vulnerable to a warming of the Earth as highly skewed sex ratios can result, potentially leading to population extinction resulting from decreased male recruitment. Recent studies have begun to quantify climate change impacts to sea turtle populations, especially in terms of predicting effects on hatchling sex ratios. However, given the inherent difficulty in studying sex ratios at this life stage, a more accurate assessment of changes in population sex ratios might be derived by evaluating the juvenile portion of foraging aggregations. We investigated the long-term trend in sex ratio of a juvenile loggerhead (*Caretta caretta*) sea turtle population inhabiting Pamlico and Core Sounds, North Carolina, USA. We used plasma testosterone reference ranges measured using radioimmunoassay (RIA) to assign sex for 959 turtles and confirmed sex assignment of a subset (N = 58) of the sampled turtles through laparoscopic examination of their gonads. Our results demonstrate that for this particular population of loggerheads, sex ratios (3Females:1Male) had not significantly changed over a 10 year period (1998–2007), nor showed any significant difference among 5-cm straight carapace length (SCL) size classes. Ultimately, these findings provide a basis for comparison with future sex ratios, and highlight the importance of establishing similar long-term studies monitoring secondary, rather than primary, sex ratios, so that needed mitigation measures to climate change impacts can be implemented.

## Introduction

Since the mid-nineteenth century, a notable increase (0.6–0.8°C) in the Earth’s mean surface temperature has been documented and is continuing at an accelerated rate [1–6]. As a result of this warming trend, changes have occurred and continue to manifest in the Earth’s biota including phenology (timing of seasonal activities) and physiology of organisms, range and distribution of species, composition of and interactions within and among communities, and structure and dynamics of ecosystems (see [4, 6] for a review). Animal and plant phenology and geographic distribution are processes most commonly monitored to assess population response to climate change. In a review of the studied phenologies of 1,598 species, it was estimated that more than half (59%) had demonstrated noticeable changes during the past 20 to 140 years [7]; earlier breeding (or first singing) of birds, flowering of plants, and spawning of amphibians are among the most common changes observed for plants and animals [7]. However, in some cases, more extreme changes can occur with these temperature increases, such as invasions of warm-water species and the eradication of other species, which can have a greater impact on populations [4]. For example, in the tropics, increases in water temperature of less than 1°C can result in large-scale coral bleaching and ensuing mortality of all coral species [4].

Likewise, for those animals more susceptible to alterations in the thermal environment, a change in climate might have more adverse effects on a population [4]. In the case of ectotherms, whose survival and reproduction are strongly dependent upon thermal conditions, climate change could have serious consequences. For many reptile species, the temperature at which the eggs are incubated determines the sex of the hatchlings, a process known as temperature-dependent sex determination (TSD) [reviewed in [8]]. During a 5-yr study monitoring air temperature and sex ratios of hatchling painted turtles (*Chrysemys picta*), empirical evidence of the sensitivity of a species with TSD to even slight changes in temperature was provided [9]. The direct relationship between annual hatchling sex ratios and mean July air temperature was documented, which in some years produced 100% male or female hatchlings. Similar to painted turtles, sea turtle female offspring are produced at higher temperatures with male offspring produced at lower temperatures [10]. Thus, if the predicted rise in global temperatures results in a disproportionate number of female hatchlings and similarly skewed adult sex ratios, single-sex populations could be produced, eventually leading to demographic collapse [9, 11, 12].

While many researchers have recognized the potential negative effect climate change could have on sea turtle populations [13], only recently have studies begun to quantify these impacts, especially in terms of predicting skewed hatchling sex ratios [14–18]. Consequently, long-term monitoring of nesting sites was recommended so that potential future changes to primary (hatchling) sex ratios could be measured [19, 20] and necessary mitigation measures implemented in a timely manner. However, primary sex ratios are influenced by a variety of factors, including geographic location of nests, time of year eggs are laid, substrate in which eggs are laid, metabolic heating, and annual weather patterns [21–26], and this variation can occur within the same beach over several years [21, 27]. Moreover, primary sex ratios are generally estimated from the sex of a limited number of dead or live hatchlings salvaged from a few nests, or inferred from measured incubation temperatures or durations [26], which requires obtaining pivotal temperatures in a laboratory setting, as well as sacrificing a sufficient number of hatchlings for validation [20]. While non-lethal methods to sex post-hatchling turtles have been developed, they are expensive and logistically difficult [28].

Alternatively, assessing the sex ratios of neritic juveniles (secondary sex ratio) might offer a better way of tracking trends in a population’s sex ratio [21, 29, 30]. Since the juvenile life stage in sea turtles is protracted, it represents many cohorts and, consequently, many years of hatchling production, incorporating sex ratio variability over time [30, 31]. Moreover, random sampling of populations is possible, as neritic juveniles are less likely to have sex-specific behavioral biases displayed by adults [21, 30]. Finally, methods used to sex juveniles are direct and non-lethal—gonads of stranded dead turtles are examined during necropsy or testosterone levels in the blood of live turtles are measured. When combined with laparoscopic verification of RIA results, blood testosterone can be an accurate means of determining sex of large numbers of juveniles [32]. Despite logistical difficulties in accessing neritic juveniles, estimated sex ratios at this stage might reveal a more accurate representation of a sea turtle population’s future reproductive potential and response to changes in the environment [21, 32].

As part of an ongoing assessment of sea turtle populations along the US Atlantic coast [33], we investigated the long-term sex ratio of neritic juvenile loggerhead (*Caretta caretta*) sea turtles inhabiting Pamlico and Core Sounds, North Carolina, USA. We measured circulating testosterone levels with radioimmunoassay (RIA) and verified sex of a subset of the sampled turtles through visual examination of their gonads to establish a range of testosterone concentrations associated with each sex. We then compared estimated sex ratios over a 10-year period (1998–2007) and among 5-cm straight carapace length (SCL) size classes to determine if significant changes in sex ratios were occurring, potentially due to changes in the climate.

## Materials and Methods

This research was conducted under the authority of U.S. Endangered Species Act Section 10(a)(1)(A) scientific research permits from the U.S. Fish and Wildlife Service (#TE-676379) and the National Marine Fisheries Service (#1260) which reviewed and approved all handling protocols for the animals in this study, including blood sampling.

### Study area and capture technique

**Fig 1:**
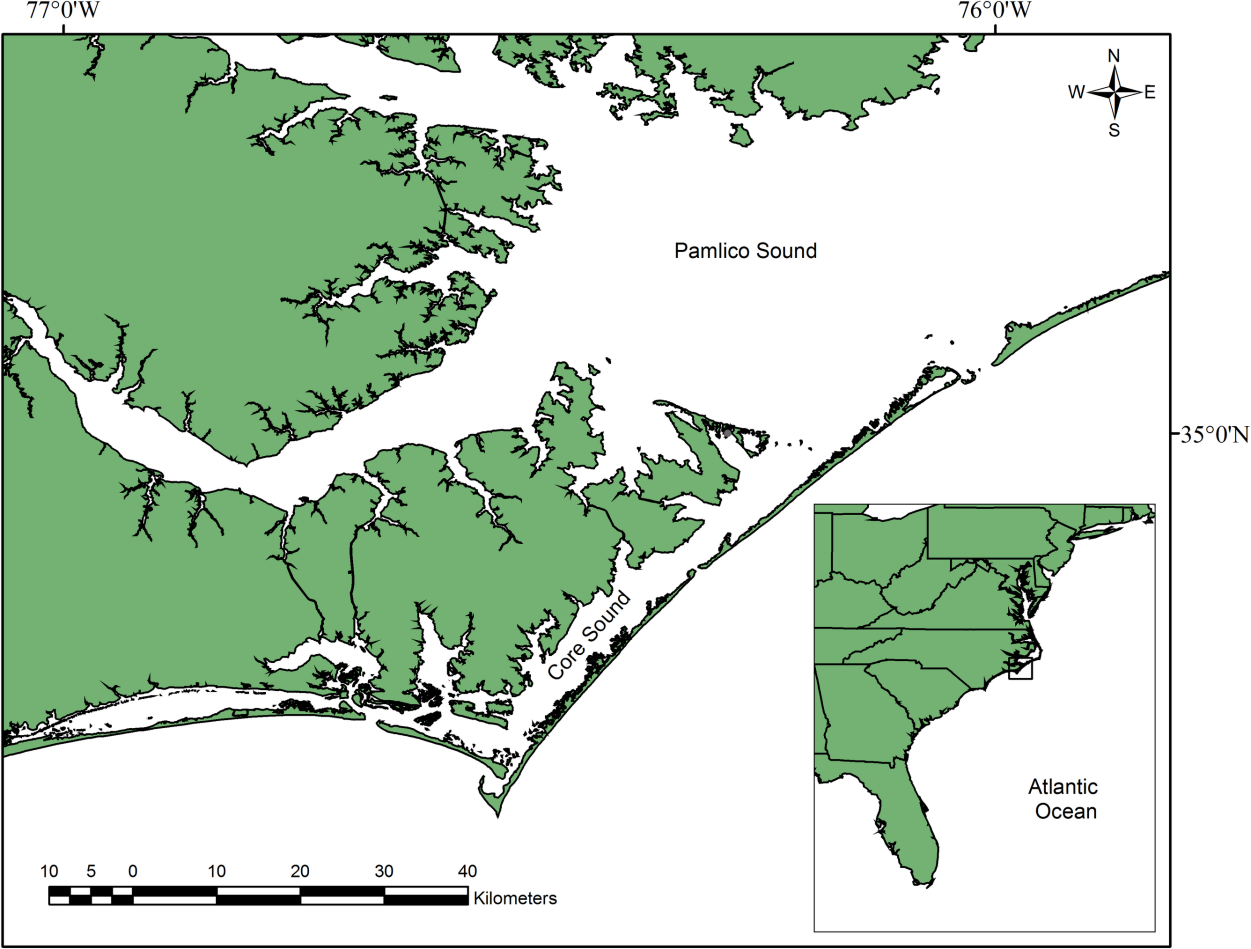
Map of study area where juvenile loggerhead (*Caretta caretta*) sea turtles were captured in pound nets and long haul seines fished in Core and Pamlico Sounds, North Carolina, USA, June to August, 1998–2007.

We sampled loggerhead turtles captured in commercial fishing gear (pound nets and long haul seines) in Core and Pamlico Sounds, North Carolina, USA (Fig 1) from 1998–2002 [34] and from 2003–2007 (this study). Pound nets are a stationary gear that passively capture targeted fish by directing them into an enclosure (pound) by means of the lead [35]. Long-haul seines are 1 km x 2 m nets pulled between two boats for up to 8 km before the catch is encircled and concentrated by pulling the net around a stake [36]. When sea turtles are also incidentally captured, they are accessible for sampling purposes. We analyzed turtles sampled only during the summer months (June–August) to increase the likelihood of sampling the resident population and to avoid any seasonal bias in testosterone concentration [34]. Using a sterile syringe with a 3.81 cm, 20 gauge needle, we collected 5 ml of blood from the dorsocervical sinus of the turtle [37]. Blood samples were immediately transferred to a sterile lithium heparin or sodium heparin tube and stored on ice for a maximum of 5 h (i.e., for the rest of the field day). We applied Inconel metal alloy size 681 self-piercing tags (National Brand and Tag Company, Newport, Kentucky, USA) to both rear flippers and injected Passive Integrated Transponder (PIT) tags (Destron-Fearing Corp., South St. Paul, Minnesota, USA, 125 kHz) in the triceps superficialis muscle of the left front flipper to recognize recaptures. We measured standard straight-line carapace length (SCL) from the nuchal notch to the posterior tip to the nearest 0.1 cm using calipers, and excluded turtles with a carapace length greater than 76 cm [30] from the analysis to prevent use of adults in this study (and avoid bias associated with various sex-specific behaviors of the adult population) [30, 31]. We recorded surface water temperature to the nearest 0.5°C using calibrated thermometers. If we encountered previously sampled turtles (from prior years), we included them, as they also were members of that year’s resident population. After returning from the field, we centrifuged blood samples for 6–10 min, then pipetted 2 ml samples of plasma into cryogenic vials, and stored them in an ultra-cold (–80°C) freezer.

### Sexing Technique

We utilized a plasma androgen sexing technique to classify sex of the turtles using a testosterone RIA procedure that was previously validated for loggerhead sea turtle plasma [30, 38, 39]. We confirmed sex for a sub-sample of turtles (N = 58) via laparoscopic examination August 2000 and July 2001 [34] and June 2004 to validate sex classification based on the RIA procedure [32]. We characterized sex by examining the shape and surface of the gonads: ovaries have an irregular shape with an undulating edge and granular surface; testes are elongated in shape with a smooth edge and surface [40]. After verifying sex, we subsequently used testosterone levels of known sex turtles to estimate plasma testosterone ranges for each sex.

### Statistical Analysis

We combined previous data (testosterone levels and laparoscopy results) collected 1998–2002 [34] with data collected 2003–2007. To determine if there was a significant difference in sex ratio (% females) among years and 5-cm size classes (SCL) ranging from 40.0–75.9 cm, we used a contingency table analysis [41]. In addition, we computed binomial confidence intervals on the proportion of females for each year and size class.

## Results

**Fig 2:**
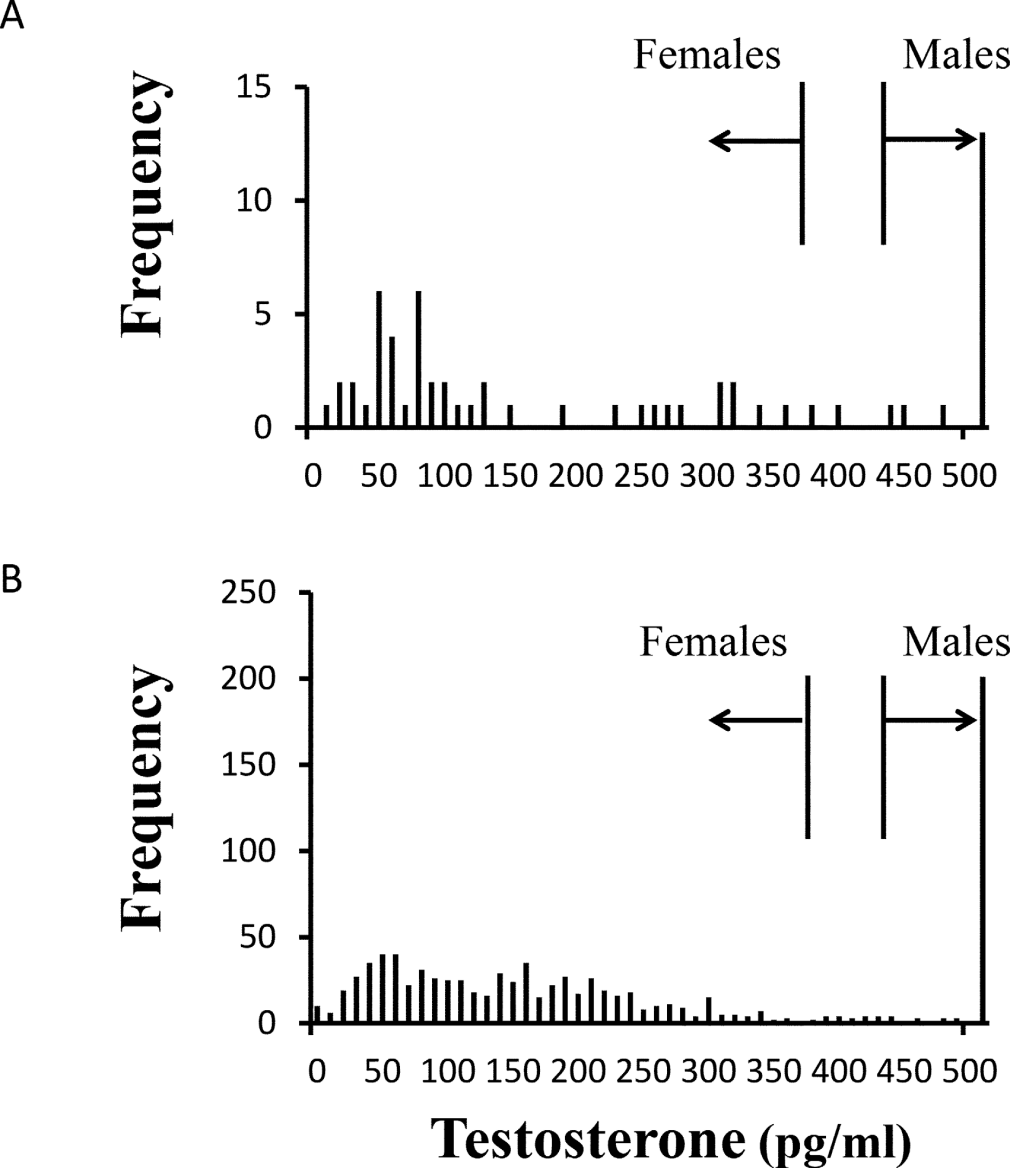
Frequency of plasma testosterone concentration of juvenile loggerhead (*Caretta caretta*) sea turtles captured in Core and Pamlico Sounds, North Carolina, USA, June to August, 1998–2007. A. that were laparoscopically examined (N = 58) (water temperatures 25–29°C) and B. that were not laparoscopically examined (N = 901) (water temperatures 20–32°C). Vertical lines indicate maximum testosterone titer of 371 pg/ml for females and minimum testosterone titer of 433 pg/ml for males. Sex could not be determined for turtles with values between vertical lines.).

We sampled blood from 959 loggerhead turtles with SCL ranging from 42.8–75.9 cm and conducted laparoscopies on 58 of them. Of the 946 whose sex was determined, 710 were female and 236 were male (Table 1). Eight turtles did not have carapace measurements, so were not included in the % females across size class analysis (Table 2). Water temperature at which turtles were sampled was 20°C or greater. Plasma testosterone concentrations for all turtles ranged from 0.1–11,420 pg/ml (N = 959). The sex ratio of laparoscoped turtles was 2.9F:1.0M. Plasma testosterone concentrations for laparoscoped females ranged from 6.7–432.0 pg/m (N = 43) while males ranged from 372.0–1884.0 pg/ml (N = 15). There was some overlap in testosterone concentration of males with females: one male (372.0 pg/ml) had a lower testosterone concentration than two of the females (394.1 and 432.3 pg/ml). Therefore, we determined that turtles having testosterone values of 371 pg/ml or less would be categorized female, turtles having testosterone values of 433 pg/ml or greater would be categorized male, and turtles having testosterone values 372–432 pg/ml would be categorized unknown (Fig 2).

**Table 1:** Number of females, males, percentage females (95% confidence intervals) and χ2 values for juvenile loggerhead (*Caretta caretta*) sea turtles.

Year	Female	Male	Total	% Female	χ2
1998	67	28	95	71 (0.60, 0.79)	1.01404
1999	63	22	85	74 (0.63, 0.83)	0.03529
2000	98	31	129	76 (0.68, 0.83)	0.06460
2001	87	30	117	74 (0.65, 0.82)	0.02564
2002	112	22	134	84 (0.76, 0.89)	5.26368
2003	108	34	142	76 (0.68, 0.83)	0.08451
2004	61	24	85	72 (0.61, 0.81)	0.47451
2005	49	17	66	74 (0.62, 0.84)	0.02020
2006	50	22	72	69 (0.57, 0.80)	1.18519
2007	15	6	21	71 (0.48, 0.89)	0.14286
Total	710	236	946		8.31051

Turtles were captured from 1998–2007 in Core and Pamlico Sounds, North Carolina, United States of America. Totals do not include unknowns (N = 13). Data from 1998–2002 are from McNeill et al. 2007.

**Table 2:** Number of females, males, percentage females (95% confidence intervals) and χ2 values across size classes (straight carapace length) for juvenile loggerheads (*Caretta caretta*).

Size Class (cm)	Female	Male	Total	% Female	χ2
40.0–44.9	3	1	4	75 (0.19, 0.99)	0
45.0–49.9	16	7	23	70 (0.47, 0.87)	0.362319
50.0–54.9	59	11	70	84 (0.74, 0.92)	3.219048
55.0–59.9	149	48	197	76 (0.69, 0.82)	0.042301
60.0–64.9	214	66	280	76 (0.71, 0.81)	0.304762
65.0–69.9	161	67	229	71 (0.67, 0.83)	2.339181
70.0–74.9	94	30	124	76 (0.67, 0.83)	0.043011
75.0–75.9*	9	3	12	75 (0.43, 0.95)	0
Total	705	233	918		6.310622

Turtles captured in Core and Pamlico Sounds, North Carolina, United States of America. Totals do not include turtles that were not measured (N = 8) or unknown sex (N = 13). Data from 1998–2002 are from McNeill et al. 2007.

* size class excludes turtles >76 cm to avoid adult behavioral biases.

**Fig 3:**
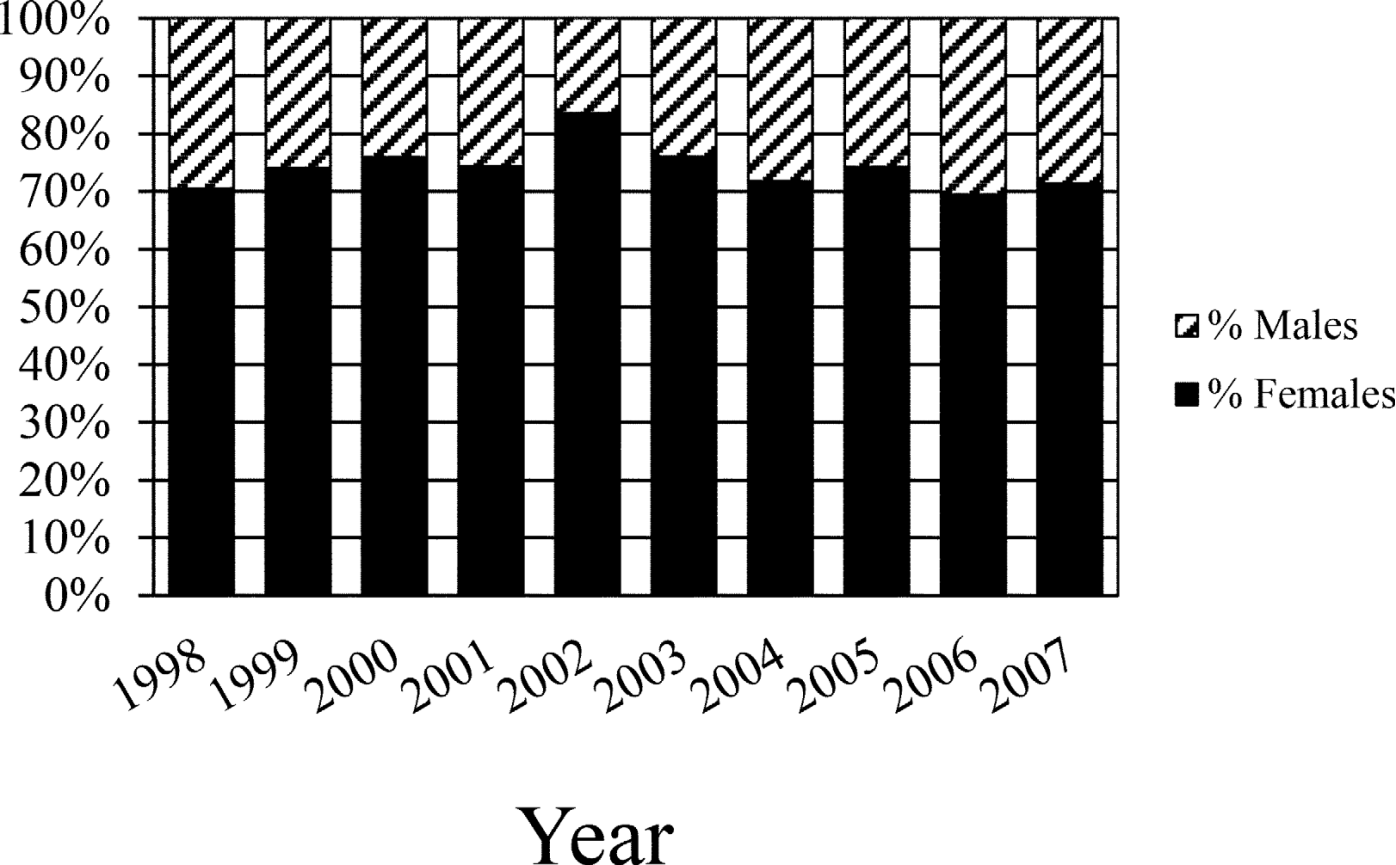
Estimated annual percentage male and female juvenile loggerhead (*Caretta caretta*) sea turtles captured in Core and Pamlico Sounds, North Carolina, USA, June to August, 1998–2007.)

We used these testosterone ranges to assign sex to turtles not laparoscoped (N = 901), resulting in 668 females, 220 males and 13 unknowns with a sex ratio of 3.0F:1.0M (Fig 2B). We did not find a significant change in sex ratios (% females) among years (χ^2^ = 8.3, df = 9, P >0.5; Table 1, Fig 3) or size classes (χ^2^ = 6.3, df = 7, P > 0.5; Table 2).

## Discussion

Despite concerns about potential changes in sex ratios of animals displaying TSD because of predicted warming conditions, during this study we found that the sex ratio of juvenile loggerhead sea turtles inhabiting North Carolina inshore waters did not change significantly from 1998–2007, nor was there a significant change in sex ratios among the different 5-cm SCL size classes (Table 2). Studies of juvenile loggerheads captured offshore south of Madeira Island (2000–2006) [42] or in the Mediterranean (2000–2011) [43] likewise did not find a significant difference among annual population sex ratios. However, a decreasing proportion of females in Madeira waters for turtles larger than the 45.0 cm SCL size class was noted [42]. Because this is the size at which turtles start leaving the pelagic habitat for neritic waters, the authors postulated that females are making the shift to neritic feeding grounds before males [42]. Although these long-term sex ratio studies do not reveal any trends over time, these data establish a baseline sex ratio for their respective areas against which results of future sex ratio analyses might be compared.

There are a number of reasons why our study’s population sex ratio may not have exhibited any noticeable trend during this time period. Given the variation in annual hatchling sex ratios [27], along with sea turtles’ long-lived, late-maturing life history, a 10 year period likely is not long enough to reveal any changes occurring in a populations’ sex ratio. Another factor to consider is that loggerheads exhibit delayed maturation, with estimates of up to 45 years [44]; thus, the data yielded by neritic juveniles are representative of what environmental conditions were like approximately 10–20 years ago [45]. Furthermore, despite overall increases in global temperatures [5], long-term mean air temperatures of coastal North Carolina from 1940–2005 actually have decreased [46]. Because of the significant relationship demonstrated between air and sea surface temperature [46], this indicates that hatchlings on these beaches may not have been exposed to warming temperatures during this time period.

In addition to representing environmental conditions from several decades ago, our population of juvenile loggerheads comprises multiple genetic nesting populations that in turn reflect different incubation environments. For instance, a study of the genetic composition of a foraging population in North Carolina, revealed that 80% of the turtles originated from the south Florida nesting population, 12% were from the northeast Florida to North Carolina nesting population, 6% from Yucatan, Mexico, and 2% from other rookeries [47]. While warmer beaches of the south Florida nesting population might be contributing a high percentage of female hatchlings to the population, the more northern and cooler nesting beaches from the northeast Florida to North Carolina nesting populations might mitigate that highly skewed female ratio by producing proportionally more male hatchlings [22]. For example, a study of the hatchling sex ratio for a North Carolina nesting beach determined a mean annual sex ratio of 58% female [46]. However, an investigation of a juvenile population of loggerheads in foraging habitat in North Carolina resulted in a more female-biased (75%) sex ratio [34]. Thus, one needs to consider the effects of climate change on the different nesting populations that contribute to the juvenile population when evaluating its sex ratio.

Conversely, a lack of a trend in our sex ratio data may indicate that loggerheads are adapting to warming temperatures. In fact, some have proposed that TSD might actually be an unexpected adaptation that could enable animals to survive warming effects of climate change [48]. For instance, nesting in different substrates, latitudes, or depths, or nesting during cooler time periods (at the beginning or end of the nesting season) could enable turtles to mitigate the harmful effects of increasing temperatures [46]. As an example, a ten day earlier median nesting date for a population of loggerheads nesting along Florida’s Atlantic coast from 1989 to 2003 was recorded [49]. Likewise, a male-biased population of red-eared sliders (*Trachemys scripta* elegans) was postulated to be the result of warming temperatures [50]. During the consequential longer nesting season, red-eared slider females laid an extra clutch when soil temperatures were relatively low, leading to the production of additional males [50]. Thus, changes in maternal behavior such as these could ameliorate some of the harmful environmental effects of climate change.

Animals with TSD can be viewed as the ‘canaries in the coal mine’ with respect to climate change [11], and these populations should be monitored to determine what effect, if any, climate change is having on sex ratios [13, 42, 51, 52], and ultimately, population viability. Many studies quantifying the effects of climate change on sea turtle population sex ratios have focused attention on monitoring hatchling sex ratios. However, considering the inherent intra- and inter-annual variation in hatchling sex ratios, the uncertainty associated with hatchling sex ratio estimation (i.e., estimates from small numbers of hatchlings or inferred from measured incubation temperatures or durations) [26], or the expense and logistical difficulty in sexing post-hatchling turtles [28], a more viable alternative would be to monitor changes in the sex ratio of the juvenile portion of the population [21, 29, 30]. Because this life stage integrates sex ratio variability over time [30, 31], allows for more random sampling of populations [21, 30], and can be sexed measuring blood testosterone levels that are verified via laparoscopy, monitoring effects of climate change at this life stage might more accurately reveal if mitigation measures to climate change are warranted [21, 32].

Despite this relevant management concern, long-term studies examining trends in secondary sex ratios for sea turtle populations are limited, with only two such studies having been conducted on juvenile loggerheads: in the Mediterranean Sea (necropsy of 271 turtles) [43] and in the northeast Atlantic (laparoscopy of 224 turtles with partial histological validation) [42]. Most other studies only look at a population’s sex ratio at a particular point in time. In contrast, our study is one of the first to examine sex ratios over a broad time span, encompassing ten years and sampling over 950 juvenile loggerhead turtles

Primary sex ratios for most loggerhead sea turtle populations, derived from nest temperatures or incubation durations [26], have been found to be strongly female-biased (see [53] for a review), with some populations in the western North Atlantic consisting of 90% or more females [53, 54]. Although still biased towards females, sex ratios of foraging juvenile loggerhead populations are not as highly skewed, displaying a 2F:1M ratio in Florida and Virginia [38], 3F:1M in North Carolina [34], 2F:1M in the northeast Atlantic [42], and 1.56F:1M in the Mediterranean [43]. However, operational sex ratios (estimated from adults actively breeding in a season) potentially appear to be more balanced [53] because male sea turtles breed more frequently than females and mate with multiple females, thus, providing a more balanced operational sex ratio despite a population’s highly skewed primary sex ratio.

Even with the existing female bias and its possible role in sea turtle life history, because sea turtles produce females during warm incubation temperatures [55], current predicted warming conditions have the potential to result in even more highly skewed sex ratios towards females, which in turn could lead to reduced genetic viability [56] and extinction [9, 57]. In a study of the potential impacts of climate change on loggerhead nesting populations, modeling exercises [46] showed that even the minimum predicted increase in air temperature (2°C) in nesting areas which are currently highly female-biased (such as southern Florida) would result in total feminization of many nests while a 3°C increase would result in many of these nests experiencing lethal incubation temperatures. However, given other environmental factors that can influence incubation temperatures (e.g., rainfall), even under an increasing temperature scenario, there will likely still be times when beach temperatures are male-producing [26].

Although sea turtles have survived past geologic temperature fluctuations, the methods used to cope with past changes and the speed with which those changes occurred is not known [58]. Current predicted climate changes are projected to happen at relatively rapid rates [5], and little empirical knowledge about the ability of sea turtles to survive these changes is available. If warming global temperatures result in detrimental changes to population sex ratios, animals with TSD could react in a number of ways–with an evolutionary response by modifying pivotal temperatures or changing to genotypic sex determination, an ecological response by altering geographic ranges or timing of nesting, or by becoming extinct [8]. Sea turtle populations are currently imperiled due to a number of anthropogenic threats including habitat alteration and loss, pollution, and incidental capture in fisheries [59]. If sea turtles are not able to adjust to impending climate changes, the result could be detrimental. Therefore, long-term monitoring of sea turtle populations should continue; however, studies should focus more on investigation of secondary, rather than primary, sex ratios, so that accurate assessments can detect changes in time for mitigation measures to be effective.

## Supporting Information

S1 Table Summary of turtle testosterone level and sex determination. (DOCX)
