# Supplementary material for: Female-Bias in a Long-Term Study of a Species with Temperature-Dependent Sex Determination: Monitoring Sex Ratios for Climate Change Research
Source: PLoS One. 2016 Aug 31;11(8):e0160911. doi: 10.1371/journal.pone.0160911 (PMC5007042; doi:10.1371/journal.pone.0160911)
Supplement: S1 Table — (DOCX) [file pone.0160911.s001.docx]

**Table S1.** Summary of turtle testosterone levels and sex determinations

| Year | Turtle ID | Temperature (⁰C) | Testosterone (pg/ml) | Sex determined via laparoscopy | Sex determined via tesosterone level |
| --- | --- | --- | --- | --- | --- |
| 1998 | 0210 | 25 |  | N | U |
| 1998 | 0600 | 27 | 166 | N | F |
| 1998 | 0601 | 26 | 85 | N | F |
| 1998 | 0605 | 28 | 60.9 | N | F |
| 1998 | 0606 | 25 | 45 | N | F |
| 1998 | 0607 | 27 | 1710 | N | M |
| 1998 | 0611 | 27 | 30.7 | N | F |
| 1998 | 0612 | 29 | 43.8 | N | F |
| 1998 | 0613 | 27 | 1280 | N | M |
| 1998 | 0615 | 27 | 489 | N | M |
| 1998 | 0616 | 25 | 633 | N | M |
| 1998 | 0687 | 22.5 | 59.4 | N | F |
| 1998 | 0690 | 29 | 23.2 | N | F |
| 1998 | 0691 | 27 | 126 | N | F |
| 1998 | 0694 | 27 | 781 | N | M |
| 1998 | 0695 | 27 | 51 | N | F |
| 1998 | 0697 | 25 | 73.4 | N | F |
| 1998 | 0698 | 24 | 975 | N | M |
| 1998 | 0700 | 28 | 67.6 | N | F |
| 1998 | 0701 | 22.5 | 37.3 | N | F |
| 1998 | 0709 | 22.5 | 33 | N | F |
| 1998 | 0711 | 22.5 | 72.4 | N | F |
| 1998 | 0715 | 27 | 11420 | N | M |
| 1998 | 0722 | 26 | 932 | N | M |
| 1998 | 0725 | 27 | 1420 | N | M |
| 1998 | 0726 | 22.5 | 33.9 | N | F |
| 1998 | 0746 | 22.5 | 457 | N | M |
| 1998 | 0748 | 27 | 79.4 | N | F |
| 1998 | 0751 | 29 | 36.6 | N | F |
| 1998 | 0755 | 27 | 104 | N | F |
| 1998 | 0756 | 27 | 1120 | N | M |
| 1998 | 0757 | 22.5 | 662 | N | M |
| 1998 | 0758 | 29 | 1540 | N | M |
| 1998 | 0760 | 28 | 150 | N | F |
| 1998 | 0761 | 27 | 56.6 | N | F |
| 1998 | 0762 | 27 | 1270 | N | M |
| 1998 | 0763 | 28 | 44.6 | N | F |
| 1998 | 0765 | 26 | 27.3 | N | F |
| 1998 | 0766 | 26 | 774 | N | M |
| 1998 | 0768 | 26 | 18.5 | N | F |
| 1998 | 0769 | 24 | 53.9 | N | F |
| 1998 | 0772 | 27 | 25.5 | N | F |
| 1998 | 0782 | 28 | 63.8 | N | F |
| 1998 | 0783 | 25 | 96.8 | N | F |
| 1998 | 0786 | 25.5 | 86 | N | F |
| 1998 | 0787 | 26 | 59.1 | N | F |
| 1998 | 0792 | 26 | 86.9 | N | F |
| 1998 | 0793 | 25.5 | 54.4 | N | F |
| 1998 | 0794 | 24 | 59.7 | N | F |
| 1998 | 0795 | 29 | 57.9 | N | F |
| 1998 | 0796 | 25 | 79 | N | F |
| 1998 | 0797 | 28 | 90.6 | N | F |
| 1998 | 0798 | 24 | 480 | N | M |
| 1998 | 0800 | 25.5 | 773 | N | M |
| 1998 | 0802 | 29 | 29.9 | N | F |
| 1998 | 0803 | 29 | 96.4 | N | F |
| 1998 | 0804 | 29 | 89.7 | N | F |
| 1998 | 0806 | 28 | 72.8 | N | F |
| 1998 | 0807 | 29 | 508 | N | M |
| 1998 | 0808 | 25 | 1020 | N | M |
| 1998 | 0810 | 27 | 54.4 | N | F |
| 1998 | 0811 | 25.5 | 85 | N | F |
| 1998 | 0812 | 28 | 88.3 | N | F |
| 1998 | 0813 | 26 | 54.4 | N | F |
| 1998 | 0814 | 26 | 78.4 | N | F |
| 1998 | 0815 | 26 | 53.1 | N | F |
| 1998 | 0816 | 26 | 55.8 | N | F |
| 1998 | 0818 | 25 | 1020 | N | M |
| 1998 | 0819 | 27 | 68.2 | N | F |
| 1998 | 0820 | 29 | 35.7 | N | F |
| 1998 | 0822 | 29 | 96.8 | N | F |
| 1998 | 0823 | 22 | 2750 |  | M |
| 1998 | 0825 | 25 | 46 | N | F |
| 1998 | 0826 | 28 | 46.5 | N | F |
| 1998 | 0827 | 29 | 109 | N | F |
| 1998 | 0828 | 28 | 1160 | N | M |
| 1998 | 0829 | 27 | 1430 | N | M |
| 1998 | 0830 | 29 | 43.4 | N | F |
| 1998 | 0831 | 27 | 35.4 | N | F |
| 1998 | 0832 | 25 | 1190 | N | M |
| 1998 | 0835 | 29 | 1670 | N | M |
| 1998 | 0836 | 25 | 66.1 | N | F |
| 1998 | 0837 | 27 | 45.7 | N | F |
| 1998 | 0839 | 27 | 51.6 | N | F |
| 1998 | 0843 | 29 | 694 | N | M |
| 1998 | 0844 | 29 | 61.4 | N | F |
| 1998 | 0845 | 29 | 40.6 | N | F |
| 1998 | 0846 | 27 | 67.7 | N | F |
| 1998 | 0849 | 27 | 111 | N | F |
| 1998 | 0855 | 27 | 5060 | N | M |
| 1998 | 1243 | 28 | 95.2 | N | F |
| 1998 | 1537 | 26 | 56.5 | N | F |
| 1998 | 1539 | 26 | 71.4 | N | F |
| 1998 | 1544 | 26 | 820 | N | M |
| 1998 | 1568 | 27 | 159 | N | F |
| 1998 | 1585 | 28 | 80.9 | N | F |
| 1999 | 0210 | 26 | 375 | N | U |
| 1999 | 0332 | 26 | 164 | N | F |
| 1999 | 0368 | 29 | 149 | N | F |
| 1999 | 0419 | 26 | 115 | N | F |
| 1999 | 0432 | 26 | 53 | N | F |
| 1999 | 0579 | 26 | 202 | N | F |
| 1999 | 0603 | 26 | 111 | N | F |
| 1999 | 0701 | 26 | 98 | N | F |
| 1999 | 0772 | 29 | 205 | N | F |
| 1999 | 0798 | 24 | 1260 | N | M |
| 1999 | 0808 | 26 | 473 | N | M |
| 1999 | 0828 | 27 |  | N | M |
| 1999 | 0842 | 27.5 | 1243 | N | M |
| 1999 | 0843 | 29.5 | 701 | N | M |
| 1999 | 0844 | 26 | 111 | N | F |
| 1999 | 0866 | 26 | 26 | N | F |
| 1999 | 0874 | 26 | 292 | N | F |
| 1999 | 0877 | 24 | 148 | N | F |
| 1999 | 0881 | 27 | 55 | N | F |
| 1999 | 0886 |  |  | N | F |
| 1999 | 0888 | 27 | 1123 | N | M |
| 1999 | 0892 | 27 | 158 | N | F |
| 1999 | 0894 | 26 | 1973 | N | M |
| 1999 | 0897 | 27.5 | 132 | N | F |
| 1999 | 0899 | 24 | 44 | N | F |
| 1999 | 0901 | 27.5 | 1210 | N | M |
| 1999 | 0903 | 27.5 | 158 | N | F |
| 1999 | 0904 | 26 | 21 | N | F |
| 1999 | 0905 | 26 | 114 | N | F |
| 1999 | 0907 | 27.5 | 513 | N | M |
| 1999 | 0908 | 26 | 23 | N | F |
| 1999 | 0910 | 29.5 | 55 | N | F |
| 1999 | 0912 | 27 | 113 | N | F |
| 1999 | 0915 | 27.5 | 97 | N | F |
| 1999 | 0916 | 26 | 118 | N | F |
| 1999 | 0917 | 29.5 | 133 | N | F |
| 1999 | 0920 | 29 | 202 | N | F |
| 1999 | 0921 | 29.5 | 77 | N | F |
| 1999 | 0923 | 27.5 | 690 | N | M |
| 1999 | 0925 | 32 | 90 | N | F |
| 1999 | 0926 | 32 | 1090 | N | M |
| 1999 | 0930 | 29.5 | 104 | N | F |
| 1999 | 0931 | 27 | 1778 | N | M |
| 1999 | 0934 | 29.5 | 2203 | N | M |
| 1999 | 0936 | 27 | 1239 | N | M |
| 1999 | 0937 | 29.5 | 142 | N | F |
| 1999 | 0938 | 29.5 | 127 | N | F |
| 1999 | 0939 | 28 | 48 | N | F |
| 1999 | 0941 | 29 | 5560 | N | M |
| 1999 | 0943 | 27 | 82 | N | F |
| 1999 | 0944 | 27 | 292 | N | F |
| 1999 | 0945 | 29 | 66 | N | F |
| 1999 | 0946 | 29 | 110 | N | F |
| 1999 | 0948 | 29 | 154 | N | F |
| 1999 | 0951 | 29 | 73 | N | F |
| 1999 | 0952 | 29 | 83 | N | F |
| 1999 | 0953 | 28 | 45 | N | F |
| 1999 | 0954 | 29 | 95 | N | F |
| 1999 | 0955 | 30 | 693 | N | M |
| 1999 | 0956 | 30 | 87 | N | F |
| 1999 | 0957 | 30 | 1520 | N | M |
| 1999 | 0958 | 30 | 187 | N | F |
| 1999 | 0960 | 30 | 73 | N | F |
| 1999 | 0961 | 30 | 318 | N | F |
| 1999 | 0962 | 30 | 144 | N | F |
| 1999 | 0963 | 30 | 1230 | N | M |
| 1999 | 0964 | 30 | 1300 | N | M |
| 1999 | 0965 | 29 | 142 | N | F |
| 1999 | 0966 | 29 | 160 | N | F |
| 1999 | 0968 | 29 | 196 | N | F |
| 1999 | 0969 | 29 | 94 | N | F |
| 1999 | 0970 | 29 | 110 | N | F |
| 1999 | 0971 | 27.5 | 70 | N | F |
| 1999 | 0973 | 27.5 | 95 | N | F |
| 1999 | 0974 | 29 | 180 | N | F |
| 1999 | 0975 | 29 | 238 | N | F |
| 1999 | 0976 | 29 | 2060 | N | M |
| 1999 | 0978 | 32 | 116 | N | F |
| 1999 | 0981 | 29 | 212 | N | F |
| 1999 | 0982 | 29 | 176 | N | F |
| 1999 | 0983 | 27 | 50 | N | F |
| 1999 | 0987 | 27 | 2040 | N | M |
| 1999 | 0989 | 27 | 84 | N | F |
| 1999 | 0990 | 29 | 200 | N | F |
| 1999 | 0991 | 29 | 210 | N | F |
| 1999 | 1044 | 29 | 2320 | N | M |
| 2000 | 0042 | 24 | 116 | N | F |
| 2000 | 0210 |  |  | N | U |
| 2000 | 0344 | 27 | 33 | N | F |
| 2000 | 0419 | 24 |  | N | F |
| 2000 | 0465 | 28 | 114 | N | F |
| 2000 | 0533 | 23 | 78 | N | F |
| 2000 | 0715 | 28 | 3702 | N | M |
| 2000 | 0746 | 28 | 1884 | M | M |
| 2000 | 0793 | 28 | 99 | F | F |
| 2000 | 0796 | 26.5 | 141 | N | F |
| 2000 | 0803 | 27 | 133 | N | F |
| 2000 | 0808 | 27 |  | N | M |
| 2000 | 0816 | 28.5 | 46 | N | F |
| 2000 | 0826 | 28 | 62 | F | F |
| 2000 | 0828 | 24 |  | N | M |
| 2000 | 0839 | 24 | 67 | N | F |
| 2000 | 0844 | 28 |  | N | F |
| 2000 | 0869 | 27 | 231 | N | F |
| 2000 | 0907 | 27 | 1228 | N | M |
| 2000 | 0938 |  |  | N | F |
| 2000 | 0946 | 29 | 126 | F | F |
| 2000 | 0956 | 27 | 67 | N | F |
| 2000 | 0975 | 23.5 | 159 | N | F |
| 2000 | 0982 | 24 | 60 | N | F |
| 2000 | 0989 | 28 | 62 | N | F |
| 2000 | 1001 | 24 | 2102 | N | M |
| 2000 | 1007 | 23 | 0.1 | N | F |
| 2000 | 1015 | 27 | 159 | N | F |
| 2000 | 1017 | 28.5 | 51 | N | F |
| 2000 | 1018 | 24 | 791 | N | M |
| 2000 | 1020 | 23 | 27 | N | F |
| 2000 | 1021 | 27 | 76 | F | F |
| 2000 | 1022 | 27 | 14 | N | F |
| 2000 | 1024 | 24 |  | N | F |
| 2000 | 1026 | 23.5 | 109 | N | F |
| 2000 | 1028 | 26.5 | 1022 | N | M |
| 2000 | 1034 | 25 | 143 | N | F |
| 2000 | 1039 | 28 | 614 | N | M |
| 2000 | 1041 | 24 | 83 | N | F |
| 2000 | 1044 | 25 | 414 | N | M |
| 2000 | 1045 | 25 | 1031 | N | M |
| 2000 | 1046 | 25 | 1110 | N | M |
| 2000 | 1048 | 28.5 | 133 | N | F |
| 2000 | 1051 | 24 | 94 | N | F |
| 2000 | 1058 | 25 | 25 | N | F |
| 2000 | 1059 | 24 | 350 | N | F |
| 2000 | 1060 | 29 | 62 | N | F |
| 2000 | 1061 | 29 | 180 | N | F |
| 2000 | 1062 | 29 | 208 | N | F |
| 2000 | 1063 | 27 | 1141 | M | M |
| 2000 | 1066 | 23 | 33 | N | F |
| 2000 | 1067 | 28 | 2236 | N | M |
| 2000 | 1070 | 27 | 643 | N | M |
| 2000 | 1071 | 27 | 12 | N | F |
| 2000 | 1073 | 27 | 129 | N | F |
| 2000 | 1074 | 27 | 47 | N | F |
| 2000 | 1075 | 26 | 723 | N | M |
| 2000 | 1076 | 25.5 | 106 | N | F |
| 2000 | 1077 | 24 | 58 | N | F |
| 2000 | 1078 | 26.5 | 14 | N | F |
| 2000 | 1079 | 26.5 | 124 | N | F |
| 2000 | 1080 | 27 | 2320 | N | M |
| 2000 | 1081 | 25 | 35 | N | F |
| 2000 | 1082 | 26 | 97 | N | F |
| 2000 | 1083 | 26.5 | 1348 | N | M |
| 2000 | 1084 | 26.5 | 71 | N | F |
| 2000 | 1085 | 26.5 | 103 | N | F |
| 2000 | 1086 | 26 | 54 | N | F |
| 2000 | 1087 | 26 | 129 | N | F |
| 2000 | 1088 | 28 | 177 | N | F |
| 2000 | 1089 | 27 | 56 | F | F |
| 2000 | 1092 | 26 | 32 | N | F |
| 2000 | 1094 | 27 | 175 | N | F |
| 2000 | 1099 | 25 | 97 | N | F |
| 2000 | 1101 | 26 | 28 | N | F |
| 2000 | 1102 | 25 | 40 | N | F |
| 2000 | 1103 | 24 | 722 | N | M |
| 2000 | 1104 | 25 | 34 | N | F |
| 2000 | 1105 | 26 | 130 | N | F |
| 2000 | 1108 | 26 | 232 | N | F |
| 2000 | 1110 | 26 | 239 | N | F |
| 2000 | 1111 | 27 | 1490 | N | M |
| 2000 | 1113 | 23.5 | 90 | N | F |
| 2000 | 1114 | 28 | 73 | F | F |
| 2000 | 1116 | 24 | 0.1 | N | F |
| 2000 | 1118 | 27 | 16 | F | F |
| 2000 | 1119 | 27 | 183 | N | F |
| 2000 | 1120 | 28 | 43 | N | F |
| 2000 | 1121 | 28 | 1383 | N | M |
| 2000 | 1122 | 21.5 | 1538 | N | M |
| 2000 | 1124 | 27 | 1282 | N | M |
| 2000 | 1125 | 27 | 530 | N | M |
| 2000 | 1126 | 28 | 128 | F | F |
| 2000 | 1127 | 28 | 648 | N | M |
| 2000 | 1129 | 21.5 | 347 | N | F |
| 2000 | 1130 | 21.5 | 196 | N | F |
| 2000 | 1131 | 28 | 59 | N | F |
| 2000 | 1132 | 28 | 18 | F | F |
| 2000 | 1134 | 28 | 101 | N | F |
| 2000 | 1135 | 27 | 43 | N | F |
| 2000 | 1136 | 27 | 57 | N | F |
| 2000 | 1137 | 27 | 568 | N | M |
| 2000 | 1147 | 26 | 90 | N | F |
| 2000 | 1156 | 24 | 33 | N | F |
| 2000 | 1159 | 25.5 | 1250 | N | M |
| 2000 | 1167 | 24 | 29 | N | F |
| 2000 | 1171 | 28 | 19 | N | F |
| 2000 | 1172 | 23.5 | 81 | N | F |
| 2000 | 1182 | 28 | 63 | N | F |
| 2000 | 1192 | 21 | 96 | N | F |
| 2000 | 1197 | 28 | 43 | F | F |
| 2000 | 1203 | 28 | 35 | F | F |
| 2000 | 1204 | 28 | 113 | F | F |
| 2000 | 1217 | 21.5 | 216 | N | F |
| 2000 | 1221 | 28 | 1323 | M | M |
| 2000 | 1224 | 24 | 79 | N | F |
| 2000 | 1226 | 21.5 | 50 | N | F |
| 2000 | 1231 | 28 | 1030 | N | M |
| 2000 | 1234 | 24 | 59 | N | F |
| 2000 | 1240 | 29 | 73 | N | F |
| 2000 | 1243 | 23 | 48 | N | F |
| 2000 | 1246 | 28 | 73 | N | F |
| 2000 | 1247 | 29 | 372 | M | M |
| 2000 | 1274 | 23 | 103 | N | F |
| 2000 | 1276 | 23 | 60 | N | F |
| 2000 | 1281 | 21 | 59 | N | F |
| 2000 | 1282 | 26 | 61 | N | F |
| 2000 | 1284 |  |  | N | F |
| 2000 | 1470 | 25 | 60 | N | F |
| 2000 | 2342 | 29 | 1537 | N | M |
| 2001 | 0029 | 26 | 258 | N | F |
| 2001 | 0112 | 27.5 | 968 | M | M |
| 2001 | 0490 | 26.5 | 72 | F | F |
| 2001 | 0686 | 29 | 980 | N | M |
| 2001 | 0746 | 28 |  | N | M |
| 2001 | 0772 | 27.5 | 55 | F | F |
| 2001 | 0798 | 28 |  | N | M |
| 2001 | 0803 | 29.5 |  | N | F |
| 2001 | 0808 | 25.5 |  | N | M |
| 2001 | 0839 | 26.5 |  | N | F |
| 2001 | 0843 | 23 | 178 | N | M |
| 2001 | 0844 | 27 |  | N | F |
| 2001 | 0923 | 29 | 952 | M | M |
| 2001 | 0956 | 29 | 50 | F | F |
| 2001 | 0974 | 27 |  | N | F |
| 2001 | 0978 | 27 |  | N | F |
| 2001 | 0982 | 27 | 105 | F | F |
| 2001 | 0989 | 24 | 0 | N | F |
| 2001 | 1044 | 26.5 |  | N | M |
| 2001 | 1051 | 23 |  | N | F |
| 2001 | 1088 | 26 |  | N | F |
| 2001 | 1090 | 28 | 60 | N | F |
| 2001 | 1094 | 30 |  | N | F |
| 2001 | 1098 | 27 | 23 | N | F |
| 2001 | 1110 | 27.5 | 52 | F | F |
| 2001 | 1115 | 27 | 6 | N | F |
| 2001 | 1138 | 27 |  | N | F |
| 2001 | 1146 | 27 | 77 | N | F |
| 2001 | 1150 |  |  | N | F |
| 2001 | 1151 | 27 | 45 | N | F |
| 2001 | 1158 | 27 | 168 | N | F |
| 2001 | 1163 | 27 | 42 | N | F |
| 2001 | 1165 | 25 | 30 | F | F |
| 2001 | 1166 | 30 | 42 | N | F |
| 2001 | 1180 | 29 |  | N | F |
| 2001 | 1184 | 27 | 16 | N | F |
| 2001 | 1185 | 27 | 31 | N | F |
| 2001 | 1192 | 27.5 |  | N | F |
| 2001 | 1193 | 27 | 583 | N | M |
| 2001 | 1198 | 27 | 434 | N | M |
| 2001 | 1201 | 27 | 20 | N | F |
| 2001 | 1203 | 30 |  | N | F |
| 2001 | 1206 | 29 | 38 | N | F |
| 2001 | 1235 | 27.5 | 445 | M | M |
| 2001 | 1243 | 25 |  | N | F |
| 2001 | 1244 | 27.5 | 601 | N | M |
| 2001 | 1250 | 29 | 51 | N | F |
| 2001 | 1251 | 28 | 752 | N | M |
| 2001 | 1252 | 29 | 53 | N | F |
| 2001 | 1266 | 28 | 854 | N | M |
| 2001 | 1268 |  |  | N | F |
| 2001 | 1269 | 26.5 | 708 | M | M |
| 2001 | 1270 | 23 | 34 | N | F |
| 2001 | 1271 | 29 | 155 | N | F |
| 2001 | 1287 | 26.5 | 410 | N | U |
| 2001 | 1288 | 27.5 | 22 | N | F |
| 2001 | 1289 | 26 | 219 | N | F |
| 2001 | 1299 | 27 | 26 | N | F |
| 2001 | 1301 | 26.5 | 969 | M | M |
| 2001 | 1302 | 29.5 | 584 | N | M |
| 2001 | 1304 | 26.5 | 837 | M | M |
| 2001 | 1305 | 28 | 28 | N | F |
| 2001 | 1307 | 25 | 48 | F | F |
| 2001 | 1308 | 25 | 471 | M | M |
| 2001 | 1309 | 29 | 76 | N | F |
| 2001 | 1310 | 27 | 33 | U | F |
| 2001 | 1311 | 25 | 50 | F | F |
| 2001 | 1314 | 27 | 15 | N | F |
| 2001 | 1315 | 29 | 2628 | N | M |
| 2001 | 1316 | 27 |  | N | F |
| 2001 | 1318 | 29.5 | 19 | N | F |
| 2001 | 1319 | 27 | 61 | N | F |
| 2001 | 1324 | 28 | 512 | N | M |
| 2001 | 1325 | 30 | 715 | N | M |
| 2001 | 1326 | 29.5 | 46 | N | F |
| 2001 | 1328 | 27.5 | 33 | N | F |
| 2001 | 1330 | 27 | 66 | N | F |
| 2001 | 1332 | 26.5 | 27 | F | F |
| 2001 | 1333 | 27.5 | 604 | N | M |
| 2001 | 1334 | 30 |  | N | F |
| 2001 | 1335 | 26 | 30 | N | F |
| 2001 | 1343 | 26 | 465 | N | M |
| 2001 | 1347 | 26 | 21 | N | F |
| 2001 | 1349 | 26.5 | 33 | N | F |
| 2001 | 1352 | 27 | 32 | N | F |
| 2001 | 1358 | 28 | 17 | N | F |
| 2001 | 1359 | 26 | 30 | N | F |
| 2001 | 1360 | 27 | 433 | N | M |
| 2001 | 1363 | 27 | 188 | N | F |
| 2001 | 1374 | 30 | 50 | N | F |
| 2001 | 1377 | 25 | 44 | F | F |
| 2001 | 1379 | 27.5 | 50 | F | F |
| 2001 | 1381 | 27 | 70 | N | F |
| 2001 | 1386 | 23 | 30 | N | F |
| 2001 | 1388 | 30 |  | N | F |
| 2001 | 1392 | 26.5 | 71 | F | F |
| 2001 | 1393 | 26.5 | 76 | F | F |
| 2001 | 1394 | 27 | 801 | N | M |
| 2001 | 1395 | 23 | 96 | N | F |
| 2001 | 1397 | 27 | 5 | N | F |
| 2001 | 1399 | 27 | 102 | N | F |
| 2001 | 1403 | 23 | 18 | N | F |
| 2001 | 1412 | 24 | 10 | N | F |
| 2001 | 1416 | 25.5 |  | N | M |
| 2001 | 1419 | 27.5 | 37 | N | F |
| 2001 | 1421 | 29 | 492 | N | M |
| 2001 | 1430 | 27.5 | 41 | N | F |
| 2001 | 1453 | 25 | 942 | N | M |
| 2001 | 1471 | 25.5 | 38 | N | F |
| 2001 | 1472 | 25 | 23 | N | F |
| 2001 | 1474 | 27 | 27 | N | F |
| 2001 | 1495 | 27.5 | 12 | N | F |
| 2001 | 1497 | 29.5 | 42 | N | F |
| 2001 | 1511 | 27 | 34 | N | F |
| 2001 | 1518 | 25 | 77 | N | F |
| 2001 | 1531 | 28 | 13 | N | F |
| 2001 | 1647 | 22 | 24 | N | F |
| 2001 | 2342 | 27 | 943 | M | M |
| 2002 | 0315 | 29 | 37.9 | N | F |
| 2002 | 0746 | 27.5 |  | N | M |
| 2002 | 0772 | 22 |  | N | F |
| 2002 | 0775 | 24.5 | 182 | N | F |
| 2002 | 0844 | 22 |  | N | F |
| 2002 | 0866 | 26.5 |  | N | F |
| 2002 | 0884 | 29 | 64 | N | F |
| 2002 | 0982 | 29 |  | N | F |
| 2002 | 0989 | 29 |  | N | F |
| 2002 | 1020 | 27.5 |  | N | F |
| 2002 | 1044 | 22 |  | N | M |
| 2002 | 1084 | 27 |  | N | F |
| 2002 | 1088 | 26.5 |  | N | F |
| 2002 | 1104 | 26.5 |  | N | F |
| 2002 | 1253 | 24.5 | 117 | N | F |
| 2002 | 1306 | 20.5 |  | N | F |
| 2002 | 1324 | 24 | 1642 | N | M |
| 2002 | 1395 |  | 0 | N | F |
| 2002 | 1426 | 22 | 49.4 | N | F |
| 2002 | 1428 | 20.5 | 131 | N | F |
| 2002 | 1457 | 27.5 | 57.9 | N | F |
| 2002 | 1505 | 24 | 119.3 | N | F |
| 2002 | 1655 | 27 | 156.1 | N | F |
| 2002 | 1690 | 24 | 37.6 | N | F |
| 2002 | 1699 | 22 | 18.8 | N | F |
| 2002 | 1701 | 27.5 | 480.1 | N | M |
| 2002 | 1724 | 22 | 65.3 | N | F |
| 2002 | 1750 | 27.5 | 133.5 | N | F |
| 2002 | 1756 | 26 | 49.3 | N | F |
| 2002 | 1763 | 24 | 42.5 | N | F |
| 2002 | 1765 | 26 | 76 | N | F |
| 2002 | 1796 | 27 | 18.6 | N | F |
| 2002 | 1798 | 24 | 1353 | N | M |
| 2002 | 1841 | 27 | 776 | N | M |
| 2002 | 1842 | 27 | 953 | N | M |
| 2002 | 1843 | 27 | 110 | N | F |
| 2002 | 1865 | 28.5 | 144.1 | N | F |
| 2002 | 1882 | 27.5 | 230 | N | F |
| 2002 | 1883 | 26.5 | 628 | N | M |
| 2002 | 1890 | 24 | 87.5 | N | F |
| 2002 | 1892 | 24 | 33.5 | N | F |
| 2002 | 1893 | 24 | 232.3 | N | F |
| 2002 | 1895 | 26.5 | 138 | N | F |
| 2002 | 1896 | 26.5 | 154 | N | F |
| 2002 | 1898 | 26.5 | 36.3 | N | F |
| 2002 | 1899 | 30 | 81 | N | F |
| 2002 | 1900 | 26.5 | 1086 | N | M |
| 2002 | 1901 | 26.5 | 39.2 | N | F |
| 2002 | 1902 | 26.5 | 2297 | N | M |
| 2002 | 1903 | 30 | 57.3 | N | F |
| 2002 | 1904 | 20.5 | 319.3 | N | F |
| 2002 | 1905 | 20.5 | 80.2 | N | F |
| 2002 | 1906 | 20.5 | 84.5 | N | F |
| 2002 | 1907 | 20.5 | 261 | N | F |
| 2002 | 1909 | 20.5 | 182.3 | N | F |
| 2002 | 1910 | 20.5 | 393 | N | M |
| 2002 | 1911 | 20.5 | 50.5 | N | F |
| 2002 | 1913 | 27 | 82.7 | N | F |
| 2002 | 1914 | 30 | 239.9 | N | F |
| 2002 | 1917 | 20.5 | 34.4 | N | F |
| 2002 | 1918 | 30 | 137.2 | N | F |
| 2002 | 1920 | 30 | 149.1 | N | F |
| 2002 | 1921 | 27 | 64.2 | N | F |
| 2002 | 1922 | 27.5 | 150.7 | N | F |
| 2002 | 1923 | 27 | 46.3 | N | F |
| 2002 | 1924 | 27.5 | 165.8 | N | F |
| 2002 | 1925 | 27.5 | 351.9 | N | F |
| 2002 | 1926 | 30 | 72 | N | F |
| 2002 | 1927 | 27 | 35.8 | N | F |
| 2002 | 1928 | 27 | 13.9 | N | F |
| 2002 | 1929 | 30 | 81.3 | N | F |
| 2002 | 1932 | 30 | 32.2 | N | F |
| 2002 | 1933 | 27 | 108.4 | N | F |
| 2002 | 1935 | 30 | 52.2 | N | F |
| 2002 | 1936 | 27 | 171.8 | N | F |
| 2002 | 1937 | 22 | 746 | N | M |
| 2002 | 1938 | 22 | 267.3 | N | F |
| 2002 | 1939 | 24 | 123 | N | F |
| 2002 | 1940 | 24.5 | 92 | N | F |
| 2002 | 1941 | 24.5 | 229.4 | N | F |
| 2002 | 1942 | 24.5 | 128 | N | F |
| 2002 | 1943 | 24.5 | 72.2 | N | F |
| 2002 | 1944 | 24.5 | 189 | N | F |
| 2002 | 1945 | 29 | 46.4 | N | F |
| 2002 | 1946 | 24.5 | 83 | N | F |
| 2002 | 1951 | 27.5 | 823 | N | M |
| 2002 | 1953 | 28.5 | 80.4 | N | F |
| 2002 | 1955 | 27 | 242 | N | F |
| 2002 | 1956 | 27 | 146 | N | F |
| 2002 | 1962 | 31 | 2179 | N | M |
| 2002 | 1967 | 23 | 1590 | N | M |
| 2002 | 1968 | 31 | 58.7 | N | F |
| 2002 | 1969 | 29 | 148.1 | N | F |
| 2002 | 1971 | 29 | 1250 | N | M |
| 2002 | 1972 | 22 | 102 | N | F |
| 2002 | 1973 | 22 | 129.4 | N | F |
| 2002 | 1974 | 22 | 55.2 | N | F |
| 2002 | 1975 | 22 | 47.8 | N | F |
| 2002 | 1976 | 22 | 117 | N | F |
| 2002 | 1977 | 22 | 3305 | N | M |
| 2002 | 1978 | 22 | 140 | N | F |
| 2002 | 1981 | 29.5 | 107 | N | F |
| 2002 | 1991 | 30 | 261.4 | N | F |
| 2002 | 1993 | 27 | 28.1 | N | F |
| 2002 | 1995 | 30 | 73.7 | N | F |
| 2002 | 2000 | 29 | 70.2 | N | F |
| 2002 | 2002 | 29 | 73.4 | N | F |
| 2002 | 2004 | 22 | 458 | N | M |
| 2002 | 2005 | 20.5 | 21.4 | N | F |
| 2002 | 2006 | 29 | 95.8 | N | F |
| 2002 | 2007 | 22 | 137.1 | N | F |
| 2002 | 2008 | 22 | 583 | N | M |
| 2002 | 2009 | 27 | 72.2 | N | F |
| 2002 | 2010 | 29 | 39.4 | N | F |
| 2002 | 2011 | 22 | 46.9 | N | F |
| 2002 | 2067 | 27.5 | 907 | N | M |
| 2002 | 2132 | 27 | 106 | N | F |
| 2002 | 2145 | 27 | 45.5 | N | F |
| 2002 | 2157 | 25.5 | 218 | N | F |
| 2002 | 2159 | 25 | 297.4 | N | F |
| 2002 | 2160 | 27.5 | 1967 | N | M |
| 2002 | 2166 | 26 | 14.6 | N | F |
| 2002 | 2168 | 27.5 | 300.9 | N | F |
| 2002 | 2184 | 26 | 135.3 | N | F |
| 2002 | 2205 | 25.5 | 187.1 | N | F |
| 2002 | 2209 | 28 | 47.7 | N | F |
| 2002 | 2214 | 27 | 33.3 | N | F |
| 2002 | 2215 | 28 | 24 | N | F |
| 2002 | 2216 | 28 | 240.5 | N | F |
| 2002 | 2234 | 27 | 5.4 | N | F |
| 2002 | 2256 | 27 | 110 | N | F |
| 2002 | 2263 | 26 | 50.6 | N | F |
| 2002 | 2279 | 27 | 131 | N | F |
| 2002 | 2295 | 27 | 825 | N | M |
| 2003 | 0315 | 29.5 | 334.9 | N | F |
| 2003 | 0490 | 28.5 | 294.3 | N | F |
| 2003 | 0686 | 27 |  | N | M |
| 2003 | 0746 | 26 | 2029 | N | M |
| 2003 | 0796 | 28 | 162.1 | N | F |
| 2003 | 0844 | 28 | 137.5 | N | F |
| 2003 | 0923 |  |  | N | M |
| 2003 | 0978 | 24 |  | N | F |
| 2003 | 1134 |  |  | N | F |
| 2003 | 1231 | 29.5 | 1357 | N | M |
| 2003 | 1334 | 25 | 325.7 | N | F |
| 2003 | 1388 | 24 | 282.6 | N | F |
| 2003 | 1416 | 26 | 1740 | N | M |
| 2003 | 1547 | 27.5 | 130.8 | N | F |
| 2003 | 1558 | 22.5 | 147.3 | N | F |
| 2003 | 1570 | 22.5 | 151.6 | N | F |
| 2003 | 1651 | 28 | 126.2 | N | F |
| 2003 | 1722 | 23 | 700.4 | N | M |
| 2003 | 1763 | 29.5 | 247.8 | N | F |
| 2003 | 1781 | 28.5 | 239.9 | N | F |
| 2003 | 1790 | 28 | 205.8 | N | F |
| 2003 | 1798 | 24 |  | N | M |
| 2003 | 1820 |  |  | N | f |
| 2003 | 1846 | 26 | 328.5 | N | F |
| 2003 | 1852 | 28.5 | 158.7 | N | F |
| 2003 | 1934 | 26 | 153.8 | N | F |
| 2003 | 1949 | 26 | 2267 | N | M |
| 2003 | 1950 | 29 | 188.7 | N | F |
| 2003 | 1951 | 28 | 1296 | N | M |
| 2003 | 1952 | 26 | 181.8 | N | F |
| 2003 | 1957 | 28.5 | 253.4 | N | F |
| 2003 | 1959 | 28.5 | 1606 | N | M |
| 2003 | 1960 | 28.5 | 324 | N | F |
| 2003 | 1961 | 28.5 | 278.2 | N | F |
| 2003 | 1963 | 28.5 | 220.6 | N | F |
| 2003 | 1967 | 27 | 2008 | N | M |
| 2003 | 1981 | 23.5 | 206.3 | N | F |
| 2003 | 1982 | 27 | 295.3 | N | F |
| 2003 | 1985 | 28 | 1404 | N | M |
| 2003 | 1986 | 28 | 118.6 | N | F |
| 2003 | 1987 | 28 | 189.7 | N | F |
| 2003 | 1988 | 22 | 189.6 | N | F |
| 2003 | 1990 | 22 | 158.2 | N | F |
| 2003 | 1996 | 21 | 144.4 | N | F |
| 2003 | 1998 | 21 | 169.4 | N | F |
| 2003 | 2001 | 22 | 292.8 | N | F |
| 2003 | 2002 | 28 |  | N | F |
| 2003 | 2003 | 24 | 500.3 | N | M |
| 2003 | 2004 |  |  | N | M |
| 2003 | 2005 | 27 |  | N | F |
| 2003 | 2008 | 21 |  | N | M |
| 2003 | 2014 | 23.5 | 2693 | N | M |
| 2003 | 2015 | 28.5 | 239.6 | N | F |
| 2003 | 2016 | 28.5 | 95.5 | N | F |
| 2003 | 2017 | 28 | 297.2 | N | F |
| 2003 | 2021 | 22 | 178 | N | F |
| 2003 | 2023 | 22 | 72.8 | N | F |
| 2003 | 2078 | 27 | 288.3 | N | F |
| 2003 | 2095 | 27.5 | 229.4 | N | F |
| 2003 | 2114 | 21 | 290.8 | N | F |
| 2003 | 2117 | 27.5 | 202.4 | N | F |
| 2003 | 2120 | 27 | 136 | N | F |
| 2003 | 2123 | 22.5 | 190.4 | N | F |
| 2003 | 2125 | 24 | 483.8 | N | M |
| 2003 | 2154 | 25 | 207.7 | N | F |
| 2003 | 2207 | 27 | 244.4 | N | F |
| 2003 | 2223 | 26 | 907.1 | N | M |
| 2003 | 2232 | 27 | 1678 | N | M |
| 2003 | 2238 | 28 | 1324 | N | M |
| 2003 | 2246 | 27 | 195.9 | N | F |
| 2003 | 2266 | 27 | 211.1 | N | F |
| 2003 | 2271 | 27 | 137.9 | N | F |
| 2003 | 2281 | 24 | 223.3 | N | F |
| 2003 | 2285 | 26 | 218.1 | N | F |
| 2003 | 2315 | 27 | 157.4 | N | F |
| 2003 | 2316 | 27 | 239.7 | N | F |
| 2003 | 2330 | 24 | 206.6 | N | F |
| 2003 | 2331 | 23.5 | 245.1 | N | F |
| 2003 | 2346 | 23 | 154.1 | N | F |
| 2003 | 2357 | 29 | 964.1 | N | M |
| 2003 | 2358 | 27 | 135.3 | N | F |
| 2003 | 2359 | 27 | 267 | N | F |
| 2003 | 2360 | 27 | 686.5 | N | M |
| 2003 | 2361 | 29 | 259.5 | N | F |
| 2003 | 2362 | 27 | 2241 | N | M |
| 2003 | 2366 | 29 | 309.6 | N | F |
| 2003 | 2367 | 27 | 158.2 | N | F |
| 2003 | 2369 | 21 | 336.5 | N | F |
| 2003 | 2370 | 24 | 1781 | N | M |
| 2003 | 2373 | 27 | 293.2 | N | F |
| 2003 | 2377 | 21 | 278.1 | N | F |
| 2003 | 2378 | 28 | 397.4 | N | U |
| 2003 | 2380 | 27 | 872.7 | N | M |
| 2003 | 2385 | 28 | 201 | N | F |
| 2003 | 2391 | 24 | 230.2 | N | F |
| 2003 | 2392 | 24 | 754 | N | M |
| 2003 | 2395 | 28 | 207.2 | N | F |
| 2003 | 2398 | 26 | 109.2 | N | F |
| 2003 | 2399 | 23.5 | 969.6 | N | M |
| 2003 | 2404 | 25 | 196.7 | N | F |
| 2003 | 2406 | 26 | 129.8 | N | F |
| 2003 | 2408 | 28 | 228.4 | N | F |
| 2003 | 2410 | 28 | 256.4 | N | F |
| 2003 | 2412 | 27.5 | 158.6 | N | F |
| 2003 | 2414 | 26 | 138.6 | N | F |
| 2003 | 2417 | 29.5 | 178.8 | N | F |
| 2003 | 2419 | 27.5 | 205.2 | N | F |
| 2003 | 2420 | 27.5 | 291.9 | N | F |
| 2003 | 2421 | 27.5 | 133.3 | N | F |
| 2003 | 2422 | 29 | 239.7 | N | F |
| 2003 | 2423 | 28 | 231.4 | N | F |
| 2003 | 2424 | 26 | 184.2 | N | F |
| 2003 | 2425 | 26 | 777.9 | N | M |
| 2003 | 2428 | 28 | 99.3 | N | F |
| 2003 | 2429 | 26 | 156.7 | N | F |
| 2003 | 2431 | 25 | 230.5 | N | F |
| 2003 | 2433 | 27.5 | 171.1 | N | F |
| 2003 | 2434 | 28 | 225.4 | N | F |
| 2003 | 2435 | 27.5 | 177.6 | N | F |
| 2003 | 2436 | 25 | 425 | N | U |
| 2003 | 2437 |  | 866.9 | N | M |
| 2003 | 2440 | 25 | 191.5 | N | F |
| 2003 | 2441 |  | 1500 | N | M |
| 2003 | 2450 | 27.5 | 201.2 | N | F |
| 2003 | 2451 | 25 | 2223 | N | M |
| 2003 | 2454 | 26 | 206.3 | N | F |
| 2003 | 2455 | 25 | 398.6 | N | F |
| 2003 | 2456 | 29 | 369.4 | N | F |
| 2003 | 2459 | 25 | 759.7 | N | M |
| 2003 | 2460 | 29 | 161.3 | N | F |
| 2003 | 2466 |  | 177.5 | N | F |
| 2003 | 2468 | 29 | 185.6 | N | F |
| 2003 | 2470 | 29 | 330.9 | N | F |
| 2003 | 2471 | 26 | 432.1 | N | U |
| 2003 | 2483 | 29 | 261.2 | N | F |
| 2003 | 2485 | 29.5 | 218.7 | N | F |
| 2003 | 2488 | 29 | 149 | N | F |
| 2003 | 2489 | 29 | 1713 | N | M |
| 2003 | 2500 | 29 | 1973 | N | M |
| 2003 | 2503 | 29 | 206.1 | N | F |
| 2003 | 2504 | 29 | 179.1 | N | F |
| 2003 | 2506 | 29 | 149.4 | N | F |
| 2003 | 2511 | 29 | 273.3 | N | F |
| 2003 | 2532 | 29 | 164.7 | N | F |
| 2003 | 4202 | 27 |  | N | F |
| 2004 | 0315 | 29 | 388 | N | F |
| 2004 | 0654 | 26.5 | 247.7 | F | F |
| 2004 | 0989 | 28 | 228.9 | N | F |
| 2004 | 1034 | 28 | 214.4 | N | F |
| 2004 | 1252 | 24 | 155.4 | N | F |
| 2004 | 1458 | 26.5 | 277.7 | F | F |
| 2004 | 1503 | 29 | 257.8 | N | F |
| 2004 | 1632 | 27 | 265.1 | N | F |
| 2004 | 1675 | 20 | 1588 | N | M |
| 2004 | 1798 | 24 | 1209 | N | M |
| 2004 | 1850 | 27 | 2251 | N | M |
| 2004 | 1861 | 28.5 | 1294 | N | M |
| 2004 | 1863 | 25 | 432.3 | F | F |
| 2004 | 1916 | 20 | 150.1 | N | F |
| 2004 | 1917 |  | 0 | N | F |
| 2004 | 1947 | 27 | 160.7 | N | F |
| 2004 | 1979 | 28 | 206 | N | F |
| 2004 | 1986 |  | 0 | N | F |
| 2004 | 2118 | 28 | 173.1 | N | F |
| 2004 | 2121 | 26 | 2344 | N | M |
| 2004 | 2131 | 28.5 | 218.6 | N | F |
| 2004 | 2141 | 27 | 216.4 | N | F |
| 2004 | 2142 | 28 | 323.9 | N | F |
| 2004 | 2150 | 25 | 222.3 | F | F |
| 2004 | 2154 | 26.5 | 255.4 | F | F |
| 2004 | 2221 | 27 | 2499 | N | M |
| 2004 | 2223 | 25 | 1556 | M | M |
| 2004 | 2225 | 25 | 355 | F | F |
| 2004 | 2232 | 28 | 0 | N | M |
| 2004 | 2259 | 26.5 | 301.5 | F | F |
| 2004 | 2265 | 25 | 318.9 | F | F |
| 2004 | 2289 | 27 | 187.3 | N | F |
| 2004 | 2292 | 28.5 | 419.6 | N | U |
| 2004 | 2296 | 27 | 3724 | N | M |
| 2004 | 2307 | 25 | 1787 | N | M |
| 2004 | 2329 | 28 | 189.5 | N | F |
| 2004 | 2346 |  | 0 | N | F |
| 2004 | 2368 | 27 | 274.4 | N | F |
| 2004 | 2379 | 28.5 | 152.3 | N | F |
| 2004 | 2396 | 28.5 | 1551 | N | M |
| 2004 | 2400 | 28 | 271.1 | N | F |
| 2004 | 2409 | 27 | 264.9 | N | F |
| 2004 | 2412 | 25.5 | 141.3 | N | F |
| 2004 | 2415 | 28 | 201.8 | N | F |
| 2004 | 2418 | 28.5 | 1459 | N | M |
| 2004 | 2422 | 25 | 0 | F | F |
| 2004 | 2430 | 25.5 | 217.6 | N | F |
| 2004 | 2432 | 25 | 2541 | N | M |
| 2004 | 2444 | 27 | 182.8 | N | F |
| 2004 | 2462 | 25.5 | 239.3 | N | F |
| 2004 | 2463 | 28 | 176.3 | N | F |
| 2004 | 2472 | 25 | 210.7 | N | F |
| 2004 | 2474 | 26 | 197.9 | N | F |
| 2004 | 2475 | 24 | 201 | N | F |
| 2004 | 2476 | 24 | 283 | N | F |
| 2004 | 2477 | 24 | 212.1 | N | F |
| 2004 | 2490 | 27 | 1190 | N | M |
| 2004 | 2491 | 27.5 | 133 | N | F |
| 2004 | 2495 | 27 | 1714 | N | M |
| 2004 | 2497 | 28.5 | 222.4 | N | F |
| 2004 | 2498 | 27.5 | 243 | N | F |
| 2004 | 2499 | 25 | 210.3 | N | F |
| 2004 | 2510 | 24 | 105.9 | N | F |
| 2004 | 2514 | 29 | 402.9 | N | U |
| 2004 | 2518 | 24 | 144.8 | N | F |
| 2004 | 2539 | 25.5 | 83 | N | F |
| 2004 | 2560 | 29 | 233.6 | N | F |
| 2004 | 2580 | 27 | 897.2 | N | M |
| 2004 | 2587 | 27.5 | 339.8 | F | F |
| 2004 | 2591 | 25 | 5022 | N | M |
| 2004 | 2625 | 27.5 | 312.6 | F | F |
| 2004 | 2635 | 28 | 1540 | N | M |
| 2004 | 2687 | 27 | 271.3 | N | F |
| 2004 | 2695 | 29 | 94.6 | N | F |
| 2004 | 2700 | 28.5 | 1455 | N | M |
| 2004 | 2702 | 24 | 133.5 | N | F |
| 2004 | 2703 | 28 | 1460 | N | M |
| 2004 | 2705 | 28 | 1691 | N | M |
| 2004 | 2710 | 28.5 | 835 | N | M |
| 2004 | 2714 | 25 | 189.2 | N | F |
| 2004 | 2726 | 25 | 149.2 | F | F |
| 2004 | 2753 | 27.5 | 185 | F | F |
| 2004 | 2789 | 25 | 394.1 | F | F |
| 2004 | 2800 | 27 | 100.4 | N | F |
| 2004 | 2802 | 27 | 1803 | N | M |
| 2004 | 3949 | 27.5 | 267.3 | F | F |
| 2004 | 4110 | 24 | 1661 | N | M |
| 2005 | 0332 | 28 | 193.3 | N | F |
| 2005 | 0842 | 28 | 0 | N | M |
| 2005 | 1051 | 24 | 144.3 | N | F |
| 2005 | 1116 | 28 | 236.6 | N | F |
| 2005 | 1195 | 29 | 225.9 | N | F |
| 2005 | 1910 | 25 | 1657 | N | M |
| 2005 | 2061 | 25.5 | 256.5 | N | F |
| 2005 | 2142 |  | 0 | N | F |
| 2005 | 2154 | 26 | 213.6 | N | F |
| 2005 | 2313 | 26 | 139 | N | F |
| 2005 | 2342 | 27 | 1195 | N | M |
| 2005 | 2377 | 29.5 | 292.3 | N | F |
| 2005 | 2397 | 31 | 186.8 | N | F |
| 2005 | 2402 | 24 | 1304 | N | M |
| 2005 | 2448 | 26 | 153.3 | N | F |
| 2005 | 2469 | 27 | 177.1 | N | F |
| 2005 | 2473 | 23 | 108.4 | N | F |
| 2005 | 2474 | 25 | 138.8 | N | F |
| 2005 | 2513 | 29 | 143.2 | N | F |
| 2005 | 2516 | 28 | 159.1 | N | F |
| 2005 | 2521 | 28 | 229.8 | N | F |
| 2005 | 2530 | 28.5 | 108.3 | N | F |
| 2005 | 2531 | 26 | 2012 | N | M |
| 2005 | 2546 | 27 | 1632 | N | M |
| 2005 | 2591 | 28 | 4212.1 | N | M |
| 2005 | 2657 | 26 | 454.7 | N | M |
| 2005 | 2661 | 26 | 1844 | N | M |
| 2005 | 2687 | 26 | 153 | N | F |
| 2005 | 2698 | 29 | 171.4 | N | F |
| 2005 | 2716 | 26 | 1246 | N | M |
| 2005 | 2727 | 29.5 | 205.1 | N | F |
| 2005 | 2739 | 28 | 304.3 | N | F |
| 2005 | 2744 | 26 | 2349 | N | M |
| 2005 | 2766 | 27 | 138 | N | F |
| 2005 | 2799 | 26 | 225.2 | N | F |
| 2005 | 2812 | 28 | 162.1 | N | F |
| 2005 | 2813 | 23 | 218.9 | N | F |
| 2005 | 2818 | 28.5 | 220.1 | N | F |
| 2005 | 2819 | 29 | 203.2 | N | F |
| 2005 | 2821 | 28 | 148.5 | N | F |
| 2005 | 2825 | 27 | 297.3 | N | F |
| 2005 | 2826 | 27 | 199.8 | N | F |
| 2005 | 2827 | 28 | 191.5 | N | F |
| 2005 | 2828 | 28 | 130.2 | N | F |
| 2005 | 2832 | 23 | 253.2 | N | F |
| 2005 | 2904 | 26 | 298.6 | N | F |
| 2005 | 2905 | 26 | 184.7 | N | F |
| 2005 | 2907 | 29.5 | 222.5 | N | F |
| 2005 | 2909 | 28 | 133.3 | N | F |
| 2005 | 2910 | 29 | 331.7 | N | F |
| 2005 | 2911 | 29 | 110 | N | F |
| 2005 | 2912 | 28 | 200.4 | N | F |
| 2005 | 2913 | 29 | 334.6 | N | F |
| 2005 | 2914 | 28.5 | 426.1 | N | U |
| 2005 | 2915 | 28 | 230.3 | N | F |
| 2005 | 2916 | 28.5 | 2304 | N | M |
| 2005 | 2917 | 25 | 1596 | N | M |
| 2005 | 2918 | 28.5 | 2095 | N | M |
| 2005 | 2919 | 29 | 1633 | N | M |
| 2005 | 2920 | 25.5 | 206.4 | N | F |
| 2005 | 2922 | 28 | 151.4 | N | F |
| 2005 | 2928 | 25.5 | 173.7 | N | F |
| 2005 | 2932 | 28 | 1428 | N | M |
| 2005 | 2933 | 25 | 1097 | N | M |
| 2005 | 2937 | 25 | 119.6 | N | F |
| 2005 | 3949 | 26 | 271.9 | Y | F |
| 2005 | 4202 | 28 | 211.6 | N | F |
| 2006 | 2022 | 22 | 207.9 | N | F |
| 2006 | 2389 | 28 | 1651 | N | M |
| 2006 | 2394 | 26 | 134.4 | N | F |
| 2006 | 2401 | 29 | 231.4 | N | F |
| 2006 | 2416 | 27 | 1891 | N | M |
| 2006 | 2426 | 25 | 1135 | N | M |
| 2006 | 2438 | 29 | 141.3 | N | F |
| 2006 | 2445 | 26 | 1794 | N | M |
| 2006 | 2447 | 25 | 99.3 | N | F |
| 2006 | 2452 | 25 | 197.9 | N | F |
| 2006 | 2458 | 26 | 280 | N | F |
| 2006 | 2493 | 26 | 184.4 | N | F |
| 2006 | 2496 | 24 | 298.5 | N | F |
| 2006 | 2501 | 23 | 269.3 | N | F |
| 2006 | 2505 | 23 | 160 | N | F |
| 2006 | 2507 | 29 | 156.8 | N | F |
| 2006 | 2508 | 26 | 198.9 | N | F |
| 2006 | 2509 | 22 | 206.5 | N | F |
| 2006 | 2515 | 23 | 125.9 | N | F |
| 2006 | 2519 | 23 | 125.9 | N | F |
| 2006 | 2529 | 29 | 2579 | N | M |
| 2006 | 2558 | 25 | 93.6 | N | F |
| 2006 | 2583 | 28 | 168.9 | N | F |
| 2006 | 2593 | 27 | 307.3 | N | F |
| 2006 | 2597 | 26 | 169.5 | N | F |
| 2006 | 2650 | 29 | 255.8 | N | F |
| 2006 | 2653 | 28 | 265.1 | N | F |
| 2006 | 2659 | 27 | 142 | N | F |
| 2006 | 2664 | 25 | 296.5 | N | F |
| 2006 | 2668 | 26 | 170.7 | N | F |
| 2006 | 2678 | 22 | 188.6 | N | F |
| 2006 | 2688 | 26 | 217.1 | N | F |
| 2006 | 2713 | 26 | 2211 | N | M |
| 2006 | 2732 | 28 | 151.9 | N | F |
| 2006 | 2733 | 23 | 637.4 | N | M |
| 2006 | 2754 | 26 | 1511 | N | M |
| 2006 | 2811 | 23 | 650.1 | N | M |
| 2006 | 2815 | 23 | 180.4 | N | F |
| 2006 | 2816 | 27 | 113.3 | N | F |
| 2006 | 2834 | 22 | 186.5 | N | F |
| 2006 | 2851 | 28 | 220.7 | N | F |
| 2006 | 2861 | 29 | 332.2 | N | F |
| 2006 | 2871 | 22 | 766.7 | N | M |
| 2006 | 2886 | 29 | 1479 | N | M |
| 2006 | 2887 | 27 | 2415 | N | M |
| 2006 | 2895 | 28 | 311.1 | N | F |
| 2006 | 2897 | 28 | 195.2 | N | F |
| 2006 | 2921 | 22 | 530.4 | N | M |
| 2006 | 2936 | 27 | 155.6 | N | F |
| 2006 | 2941 | 26 | 1191 | N | M |
| 2006 | 2943 |  | 1204 | N | M |
| 2006 | 2952 | 28 | 183.7 | N | F |
| 2006 | 2970 | 22 | 179.3 | N | F |
| 2006 | 2972 | 29 | 219.7 | N | F |
| 2006 | 2973 | 28 | 1449 | N | M |
| 2006 | 3001 | 27 | 417.3 | N | U |
| 2006 | 3005 | 26 | 186.8 | N | F |
| 2006 | 3006 | 27 | 197.4 | N | F |
| 2006 | 3011 | 29 | 160.7 | N | F |
| 2006 | 3012 | 29 | 804.6 | N | M |
| 2006 | 3013 | 29 | 187.4 | N | F |
| 2006 | 3031 | 28 | 193.8 | N | F |
| 2006 | 3033 | 28 | 256.7 | N | F |
| 2006 | 3034 | 28 | 1326 | N | M |
| 2006 | 3035 | 28 | 408.7 | N | U |
| 2006 | 3036 | 28 | 280.2 | N | F |
| 2006 | 3039 | 29 | 1619 | N | M |
| 2006 | 3042 | 29 | 2185 | N | M |
| 2006 | 3043 | 29 | 109.6 | N | F |
| 2006 | 3051 | 28 | 135.3 | N | F |
| 2006 | 3059 | 28 | 3168 | N | M |
| 2006 | 3101 | 24 | 922.8 | N | M |
| 2006 | 3113 | 24 | 209.2 | N | F |
| 2006 | 3142 | 24 | 91.6 | N | F |
| 2007 | 0746 | 24 | 1094 | N | M |
| 2007 | 0772 | 27 | 167.9 | N | F |
| 2007 | 1270 | 26 | 160.6 | N | F |
| 2007 | 1458 | 26 | 139.3 | N | F |
| 2007 | 1916 | 26 | 263.5 | N | F |
| 2007 | 2154 | 24 | 177.3 | N | F |
| 2007 | 2390 | 26 | 158.2 | N | F |
| 2007 | 2886 | 28 | 1716 | N | M |
| 2007 | 2915 | 28 | 100 | N | F |
| 2007 | 2952 | 28 | 151.9 | N | F |
| 2007 | 2972 | 28 | 155.8 | N | F |
| 2007 | 2994 | 23 | 84 | N | F |
| 2007 | 3031 | 24 | 126.2 | N | F |
| 2007 | 3033 | 24 | 0 | N | F |
| 2007 | 3084 | 23 | 999.1 | N | M |
| 2007 | 3145 | 26 | 1223 | N | M |
| 2007 | 3238 | 26 | 1751 | N | M |
| 2007 | 3290 | 25 | 159.2 | N | F |
| 2007 | 3414 | 23 | 173 | N | F |
| 2007 | 3419 | 28 | 429.4 | N | U |
| 2007 | 3440 | 29 | 119.1 | N | F |
| 2007 | 3461 | 24 | 605.7 | N | M |
